# Effect of Irrigation, Bur Size and Rotational Speed on Thermographic Heat at Implant Site Osteotomy Interface. An In Vitro Study

**DOI:** 10.1111/clr.14462

**Published:** 2025-06-16

**Authors:** Karl Paeßens, Leonard van Bebber, Holger Zipprich, Paul Weigl

**Affiliations:** ^1^ Department of Prosthodontics Faculty of Oral and Dental Medicine at Goethe University Frankfurt am Main Germany; ^2^ Dental Office PaeßensZahnwelten Kleve Germany; ^3^ Freelance Engineer Darmstadt Germany

**Keywords:** bone‐implant interface, cortical bone, hot temperature, immediate dental implant loading, osteotomy, thermography

## Abstract

**Objectives:**

This study aimed to evaluate the impact of drill diameter, rotational speed, and irrigation on critical heat generation (≥ 47°C) at the dynamic bone‐drill interface during dental implant osteotomy in pre‐existing pilot bone cavities.

**Material and Methods:**

Bone samples were cut such that immediate and direct thermographic measurements at the dynamic bone‐drill interface were possible. Osteotomy cavities of 2.4 mm width were expanded to either 3.2 or 3.8 mm in cortical bovine bone with a thickness of 3.5 mm, using two‐bladed twist drills at rotational speeds of 200, 600, or 1000 rpm, with or without saline irrigation. A logistic regression model was developed to predict the likelihood of reaching temperatures ≥ 47°C during osteotomy based on these parameters.

**Results:**

The absence of irrigation, major osteotomy diameter expansion, and higher rotational speeds were identified as significant risk factors for increasing the bone‐drill interface temperature by more than 10°C (OR: irrigation 177.53; expansion step 9.94; speed by 400 rpm 4.94). No osteotomy performed at a low rotational speed (200 rpm) resulted in a critical temperature rise in either drill diameter group when irrigation was provided. However, temperatures exceeded 47°C across all groups when irrigation was absent.

**Conclusions:**

Dental implant osteotomy procedures without irrigation result in critical heat stress at the bone‐drill interface, even at low drilling speeds. Shortened protocols with large drill diameter differences of up to 1.4 mm can be safely implemented when drilling at 200 rpm with irrigation. Osteotomy protocols can therefore be shortened while maintaining safety.

**Trial Registration:**

No clinical trial was included in the study

## 1 Introduction

Immediately loaded implants represent a patient‐oriented therapeutic option that can be offered to an expanding patient population, facilitated by advancements in special implant macro‐designs and osteotomy protocols. These designs and protocols aim to achieve high primary stability while minimizing damage to the osteotomy cavity walls by reducing the dead zone of osteocytes at the implant interface caused by bone trauma (Chen et al. 2018). The dead zone deteriorates before new bone formation occurs, leading to implant loosening depending on its size and extent (Marzook et al. 2020). While preparing the implant site (osteotomy), friction between drill and bone surface generates heat at the bone‐drill interface, which affects bone vitality. Bone vitality and osseointegration are negatively affected by heating inserted implants at ∆10°C above body temperature (~47°C) for ≥ 1 min (Eriksson and Albrektsson 1983), and this value has been established as the limit for temperatures leading to potential harmful heat stress (Augustin et al. 2009; Augustin, Davila, et al. 2012a; Delgado‐Ruiz et al. 2018; Kirstein et al. 2016; Lajolo et al. 2018; Lucchiari et al. 2016; Raj et al. 2021). If the osteotomy results in a temperature increase > 10°C in the bone, the dead zone will increase, the implant stability will decrease, and lead to an increased risk of the implant being prematurely lost (Berman et al. 1984; Schmelzeisen 1992; Heuzeroth et al. 2021; Leunig and Hertel 1996; Trisi, Berardini, Falco, Podaliri Vulpiani, and Perfetti 2014a; Trisi, Berardini, Falco, Vulpiani, and Masciotra 2014b).

Manufacturers of dental implants provide recommendations regarding drilling parameters, such as drill speed and expansion steps. However, these guidelines are not consistently supported by evidence from scientific studies, and the existing literature presents contradictory findings.

The correlation of drill rotation speed on increasing temperature has been described as inverse, that is, faster speed decreases heat stress (Bogovic et al. 2016; Kirstein et al. 2016; Marzook et al. 2020), and positive, that is, slower speed decreases heat stress (Delgado‐Ruiz et al. 2018; Fraguas de San José et al. 2020; Raj et al. 2021). The correlation of the increase in drill diameter is also described as inverse and positive, meaning a smaller increase in drill diameter has been associated with higher (Mihali et al. 2018; Raj et al. 2021) but also with lower (Bacci et al. 2019; Frösch et al. 2019) temperatures.

Bone layers removed by drilling prior to final insertion of an implant do not affect osseointegration. As extension drilling is necessary to achieve the final dimension following pilot drilling with narrow diameters, the temperature increase during the final drilling is of particular importance. This is emphasized by the findings of Gehrke (2015), who found a negative effect on the histological composition of the bone when a worn final drill was used instead of an unused one.

An experimental setup capable of directly measuring the bone‐drill interface during an irrigated expansion osteotomy has not yet been established, despite being essential for realistically estimating temperature development (Harder et al. 2018; Zipprich et al. 2019). This study aimed to develop such a setup to evaluate the effects of drill diameter, rotational speed, and irrigation on temperature at the bone‐drill interface during osteotomy preparation. The hypothesis was that a combination of low rotational speed and small increments in osteotomy diameter would prevent the bone temperature from exceeding the critical threshold of 47°C, even in the absence of irrigation (Bernabeu‐Mira et al. 2024; Calvo‐Guirado et al. 2015).

## 2 Material and Methods

### 2.1 Experimental Summary

We expanded 120 pre‐existing 2.4‐mm pilot bone cavities to final diameters of 3.2 (small step—SS) or 3.8 mm (large step—LS) using twist drills at rotational speeds of 200, 600, or 1000 rpm, with (with irrigation—WI) or without irrigation (no irrigation—NI). Bone‐drill interface temperatures at the moment of perforation were recorded using a thermographic camera positioned on the opposite side.

### 2.2 Specimens

We simulated the human mandible using the distal regions of bovine ribs (Aerssens et al. 1998) with a cortical thickness (7 in Figure 2) of 3.5 ± 0.05 mm. Ethics approval was not required for this in vitro study. The ribs obtained from a slaughterhouse were kept moist and frozen on the day of deboning (Calvo‐Guirado et al. 2015; Mihali et al. 2018; Sedlin and Hirsch 1966; Sumer et al. 2011). A 2.4‐mm diameter cavity was prepared without perforating the specimen and heated to body temperature in a water bath before the measurements were taken. Additional precautions based on the measurement method are described in the Specimen Preparation subsection.

### 2.3 Test Groups

**FIGURE 1:**
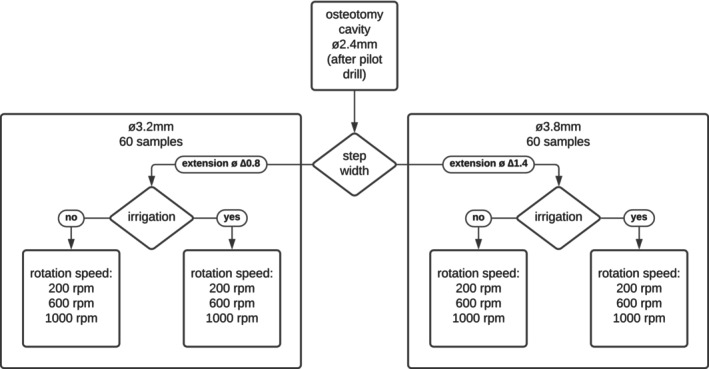
Flowchart of test groups and samples.

**FIGURE 2:**
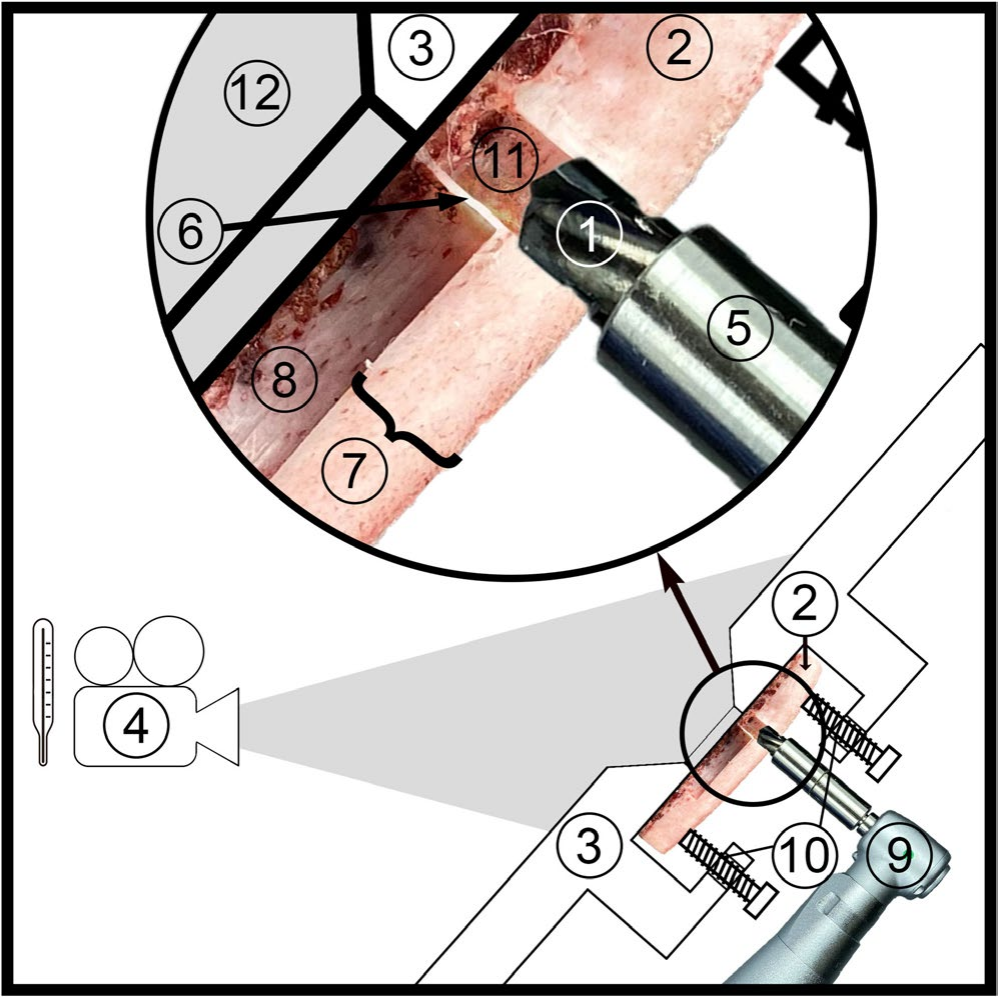
Experimental system. (1) Drill, (2) bone plate, (3) aluminum plate, (4) thermographic camera, (5) drill stop, (6) lamella, (7) compacta thickness (3.5 mm), (8) recess, (9) handpiece, (10) fastening screws, (11) pilot hole, (12) funnel‐shaped recess.

Pilot holes with a diameter of 2.4 mm were enlarged by 0.8 and 1.2 mm at 200, 600, and 1000 rpm, both with and without external irrigation with saline at 56 mL/min at room temperature (Mercan et al. 2019; Mihali et al. 2018). Double twist parallel step drills, without “relief angele” according to Chacon et al. (2006), with diameters of 3.2/3.6 and 3.8/4.2 mm (Nobel Biocare Services AG, *Zürich, Swit*z*erland*) were fitted with a stop at 4.3 mm (5 in Figure 2) such that only the smaller diameter (3.2 or 3.8 mm) would have an effect during osteotomy. This resulted in 12 test groups: 6 using 3.2/3.6 mm drills and 6 using 3.8/4.2 mm drills. Each drill was used once for each corresponding group (6 times) (Figure 1, Table 1).

**TABLE 1:** Parameters, mean, and standard deviations of 12 test groups.

Group	Irrigation	Drill ø (mm)	Δ ø (mm)	Speed (rpm)	Average max temp (°C)	± SD (°C)
**1 (SS‐WI‐200rpm)**	WI	3.2/3.6	0.8	200	**38.08**	1.01
**2 (SS‐WI‐600rpm)**	WI	3.2/3.6	0.8	600	**43.18**	2.92
**3 (SS‐WI‐1000rpm)**	WI	3.2/3.6	0.8	1000	**45.28**	3.56
**4 (LS‐WI‐200rpm)**	WI	3.8/4.2	1.4	200	**38.81**	1.93
**5 (LS‐WI‐600rpm)**	WI	3.8/4.2	1.4	600	**45.97**	3.93
**6 (LS‐WI‐1000rpm)**	WI	3.8/4.2	1.4	1000	**50.06**	4.38
**7 (SS‐NI‐200rpm)**	NI	3.2/3.6	0.8	200	**50.31**	3.45
**8 (SS‐NI‐600rpm)**	NI	3.2/3.6	0.8	600	**54.39**	4.69
**9 (SS‐NI‐1000rpm)**	NI	3.2/3.6	0.8	1000	**66.89**	26.57
**10 (LS‐NI‐200rpm)**	NI	3.8/4.2	1.4	200	**54.00**	4.46
**11 (LS‐NI‐600rpm)**	NI	3.8/4.2	1.4	600	**59.94**	6.32
**12 (LS‐NI‐1000rpm)**	NI	3.8/4.2	1.4	1000	**66.78**	15.33

Abbreviations: LS, large step (2.4–3.8 mm ø); max temp, maximal temperature; NI, no irrigation; ø, diameter; SD, standard deviation; SS, small step (2.4–3.2 mm ø); WI, with irrigation; Δ, difference.

### 2.4 Specimen Preparation

The bone samples required pilot cavities impermeable to water and a recess on the opposite side to allow direct and immediate measurement of the bone‐drill interface when the sample was perforated due to pilot cavity expansion. For this purpose, bone plates (2 in Figure 2), approximately 30 × 40 mm^2^ in size, were cut from the convex side of the ribs. In cortical bone with a thickness exceeding 5.0 mm, 5‐mm‐deep holes (11 in Figure 2) with a diameter of 2.4 mm were drilled using a KaVo MASTERsurg surgical unit (9 in Figure 2) (KaVo Dental GmbH, Biberach, Germany). On the opposite side of the specimen, a 10‐mm‐diameter recess (8 in Figure 2) was milled into the bone plate to serve as a window for the thermographic camera and to standardize the cortical bone thickness (7 in Figure 2) to 3.5 ± 0.05 mm. The recess was positioned close to the pilot cavity, leaving a cortical bone lamella (6 in Figure 2) with a thickness of 0.3 mm or 0.6 mm (corresponding to test groups with cavity expansions to diameters of 3.2 mm and 3.8 mm, respectively). The lamella was intentionally designed to be laterally perforated during the extension of the pilot cavity.

### 2.5 Procedure

The test specimens were fixed on an aluminum plate (3 in Figure 2) and heated to 38°C in a water bath for approximately 40 s. The specimens were monitored in the aluminum housing using a thermographic camera (4 in Figure 2) integrated into the measuring device. Once the bone temperature reached body temperature (36.6°C) (Obermeyer et al. 2017), the pilot cavity (11 in Figure 2) was expanded freehand from the crestal side using a surgical handpiece (9 in Figure 2) with uniform force. The thermographic camera (4 in Figure 2) recorded video data at 125 frames per second throughout the entire expansion drilling process. The maximum temperature of the drill‐bone interface at the moment of drill penetration was determined from the recorded data.

### 2.6 Setup

**FIGURE 3:**
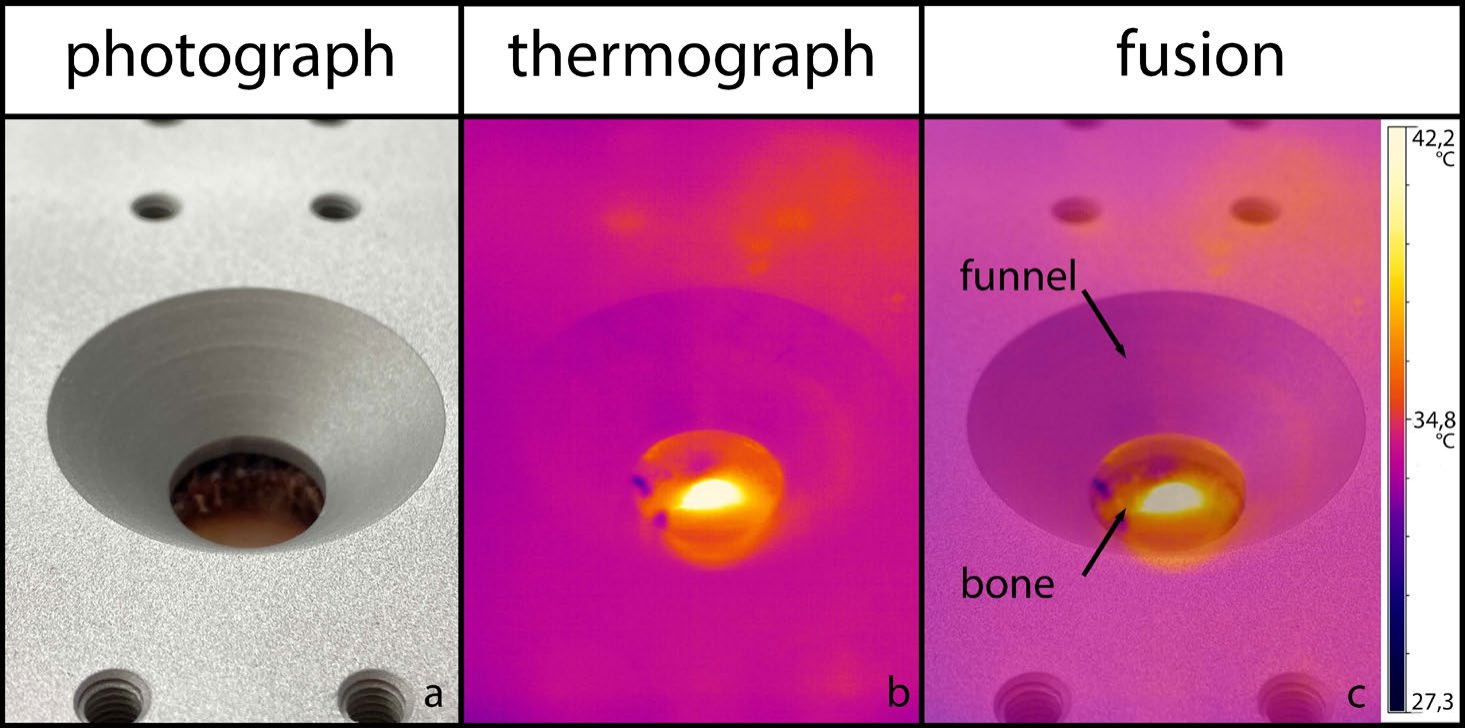
Imaging and. (a) Light image of measuring point from perspective of thermographic camera. (b) Thermal image of drill at moment of penetration. (c) Projection of light image (a) onto thermal image (b) with labeling.

Figure 2 illustrates the experimental setup. An Optris Pi 640 thermographic camera (Optris GmbH, Berlin, Germany) (4 in Figure 2), with a resolution of 640 × 480 pixels, a sensitivity of 0.1°C, and an absolute resolution of ±2°C, was positioned at a 45° angle and a 10 cm distance from the recess of the bone sample (2 in Figure 2). The bone specimens (2 in Figure 2) were securely screwed to an aluminum plate (3 and 10 in Figure 2). To minimize external thermal interference and simulate the oral cavity's thermal radiation, the entire setup was enclosed in an aluminum housing. Figure 3a highlights the funnel‐shaped recess (12 in Figure 2) incorporated into the aluminum plate (3 in Figure 2). The recess in the cancellous bone (8 in Figure 2) of the bone plate (2 in Figure 2) ensured that the lamella (6 in Figure 2) was directly visible from the thermographic camera's perspective.

### 2.7 Statistical Analysis

Data were analyzed using the readxl, ggplot2, dplyr, and datasets packages in R (R Foundation for Statistical Computing, Vienna, Austria). Test results with temperatures above 47°C were classified as critical. A multivariate logistic regression model was developed to determine the probability of exceeding a maximum temperature of 47°C as a function of irrigation, rotation speed, and step size. Logistic regression models calculate probabilities based on parameter‐specific coefficients, where higher coefficients indicate stronger influences. To ensure the significance of these influences was assessed using *p*‐values, with *p* ≤ 0.05 considered statistically significant. The model's intercept, which calibrates the overall prediction, was not interpreted, as it lies outside the scope of this study. To ensure model stability and power, the number of events per predictor was set between 10 and 20 (Peduzzi et al. 1996).

### 2.8 Preliminary Wear Test

We compared the temperatures of 10 unused drills with those of a single drill used 10 times in succession during a preliminary test series with the parameters of group 12 (LS‐NI‐1000rpm). Osteotomies were consistently performed at two adjacent sites approximately 40 mm apart on the same rib to minimize distortions.

## 3 Results

### 3.1 Preliminary Test Series

The frequency of reuse and the temperature at the bone‐drill interface showed no significant correlation (*p* = 0.8837; Pearson product–moment correlation). Additionally, no significant difference was observed between the final group 12 (LS‐NI‐1000rpm, 6th application) and the pre‐test group using new drills (*p* = 0.8501; Student's *t*‐test). Therefore, reusing the same drills up to six times did not affect temperature increments in this series.

### 3.2 Main Test

**FIGURE 4:**
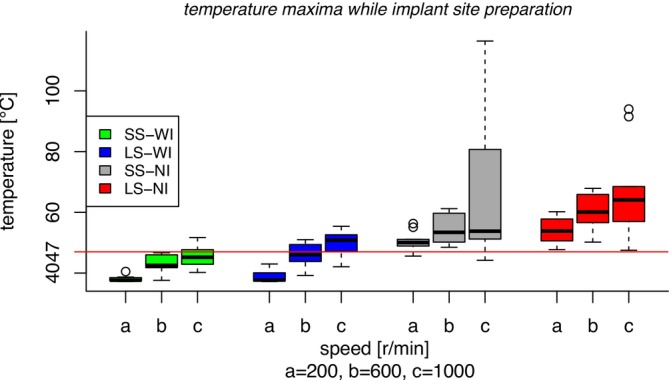
Box plot of maximum temperatures. LS, large step (2.4–3.8 mm ø); NI, no irrigation; SS, small step (2.4–3.2 mm ø); WI, with irrigation.

The maximum temperatures recorded during expansion osteotomies with irrigation and without irrigation were 55.4°C and 116.4°C, respectively (1000 rpm). For drill speeds of 600 rpm, the maximum temperatures were 51°C with irrigation and 67°C without irrigation. At a drill speed of 200 rpm, temperatures of 43°C with irrigation and 60°C without irrigation were measured. The means and standard deviations of the maximum temperatures are shown in Table 1 and Figure 3, while Figure 4 illustrates the distribution of measurements for each group in a box plot diagram. In contrast to all other groups, temperatures in groups 1 (SS‐WI‐200rpm), 2 (SS‐WI‐600rpm), and 4 (LS‐WI‐200rpm) never exceeded 47°C. In group 3 (SS‐WI‐1000rpm), approximately 40% of the recorded values surpassed this threshold, while in group 5 (LS‐WI‐600rpm), the proportion was 50%. In groups 6 (LS‐WI‐1000rpm), 7 (SS‐NI‐200rpm), and 9 (SS‐NI‐1000rpm), 80% of the values exceeded 47°C, and in groups 8 (SS‐NI‐600rpm), 10 (LS‐NI‐200rpm), 11 (LS‐NI‐600rpm), and 12 (LS‐NI‐1000rpm), all measured values were above this critical temperature.

### 3.3 Irrigation

Irrigation was a significant predictor of the probability of exceeding the critical temperature of 47°C (*p* = 0.00092). The critical temperature was exceeded in 17 out of 60 tests with irrigation, compared to 56 out of 60 tests without irrigation. The odds of exceeding the temperature threshold were 177.53 times higher when irrigation was not applied (OR = 177.53; 95% CI: 35.67–1448.46).

### 3.4 Expansion Step

An increase in the drill diameter from 0.8 to 1.4 mm significantly altered the probability of exceeding the critical temperature of 47°C (*p* = 0.0012). Expanding the cavity by 1.4 mm resulted in a critical temperature rise in 13 out of 30 tests compared to 4 out of 30 tests with a 0.8 mm expansion when irrigation was provided. Without irrigation, 30 out of 30 tests resulted in a critical temperature rise when expanded by 1.4 mm and 26 out of 30 when expanded by 0.8 mm. The odds of exceeding the temperature threshold were 9.94 times higher when the drill diameter was increased from 0.8 to 1.4 mm (OR = 9.94; 95% CI: 2.76–46.50).

### 3.5 Drill Speed

Rotation speed significantly influenced the odds of exceeding the critical temperature of 47°C (*p* = 0.00049). The temperature exceeded 47°C in zero out of 20 tests at 200 rpm, 5 out of 20 tests at 600 rpm, and 12 out of 20 tests at 1000 rpm when irrigation was provided. Without irrigation, 18 out of 20 tests at 200 rpm, 20 out of 20 tests at 600 rpm, and 18 out of 20 tests at 1000 rpm resulted in a critical temperature rise. Increasing the speed by 400 rpm led to 4.94 times higher odds of exceeding the critical temperature (OR = 4.94; 95% CI: 2.18–13.54).

### 3.6 Model Results

**FIGURE 5:**
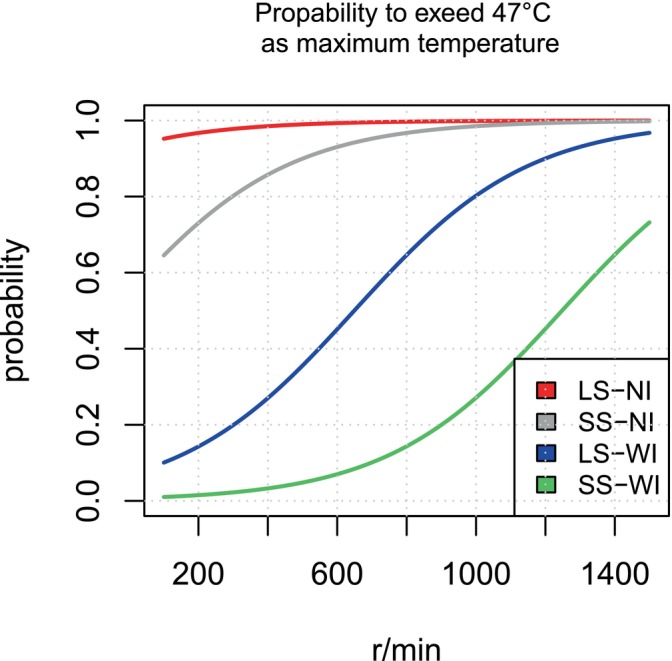
Probability (W) of reaching maximum temperatures above 47°C as a function of irrigation, rotation speed, and step size. LS, large step (2.4–3.8 mm ø); NI, no irrigation; SS, small step (2.4–3.2 mm ø); W, probability; WI, with irrigation.

With three categorical predictors and 73 positive events out of 120 samples, the number of negative events is 47. The EPV (Events per predictor) is calculated as (47 events ÷ 3 predictors = 15.7), which falls within the recommended range of 10 to 20 events per predictor to ensure model stability and power (Austin and Steyerberg 2017). Table 2 displays the coefficients of the individual parameters obtained from the multivariate logistic regression model, along with the resulting formula. Figure 5 visualizes this model by plotting the probability of exceeding the critical temperature as a function of rotation speed for all four combinations of step size and irrigation. For instance, in the large step with irrigation group (LS‐WI), the probability of a critical temperature increase was estimated to be 0.9 (90%) at a rotation speed of 1400 rpm and below 0.2 (20%) at 200 rpm, as indicated by the blue curve. The model predicts that low rotational speeds and small drill diameter increments will not prevent critical temperature development when irrigation is absent, as shown by the grey curve. Based on these findings, the study hypothesis must be rejected.

**TABLE 2:** Multivariate logistic regression analysis to predict maximum temperatures ≥ 47°C.

Parameters	Coefficient (CI 2.5; CI 97.5)	Standard Error	Odds Ratio (CI 2.5; CI 97.5)	*p*
Irrigation (off vs. on)	5.179 (3.57; 7.28)	0.93	177.53 (35.67; 1448.46)	0.00092
Expansion step size (Δ1.4 vs. 0.8)	2.297 (1.01; 3.84)	0.71	9.94 (2.76; 46.50)	0.00122
Speed/400 rpm	1.596 (0.78; 2.61)	0.46	4.94 (2.18; 13.54)	0.00049
Intercept	−4.984 (−7.48; −3.04)	1.12		0.02062

*Note:* Formula *F*: Probability (*W*) of reaching maximum temperatures > 47°C = 1/(1 + e^−z^), where z = 1.596 (rotation speed in 400 rpm) + 5.179 (if unirrigated) + 2.297 (if step = 1.4 mm) −4.984 (intercept). The presented coefficients may only be interpreted within the multivariate logistic regression model and do not describe any linear regression.

## 4 Discussion

The study was motivated by the need to develop a reliable method for measuring heat generation during dental implant cavity expansion, particularly when irrigation is applied. A robust in vitro setup must measure the temperature directly at the bone‐drill interface, where heat is primarily generated (Zipprich et al. 2019). However, thermocouples cannot be positioned within the dynamic bone‐drill interface due to the mechanical nature of the process. Although thermographic methods offer a solution for measuring this interface, they must provide direct and immediate insight to capture accurate temperature data. Systems relying on thermocouples or lacking immediate thermographic insight tend to underestimate the actual peak temperature. For example, a thermocouple placed 1 mm away from a heat source in a finite element model of simulated mandibular cortical bone recorded temperatures that were 19.4°C lower than the heat source itself (Stocchero et al. 2019). In contrast, thermographic methods have demonstrated greater accuracy in temperature measurement compared to thermocouples (Chakraborty et al. 2023; Harder et al. 2018). To ensure accuracy, infrared thermometers must be categorized into two types: thermographic cameras and pyrometers. Pyrometers provide a single temperature value for a measured area, whereas thermographic cameras produce an infrared image in which each pixel corresponds to a specific temperature value. In areas containing both hot and cold spots, pyrometers calculate an average temperature for the entire area, whereas thermographic cameras are capable of distinguishing the maximum temperature from the surrounding values (Amazon 2021). Consequently, pyrometers tend to underestimate the maximum temperature when compared to thermographic cameras, which provide a more accurate representation of localized heat distribution. However, in many studies, the thermographic setup did not directly record the bone‐drill interface, but a surface of the surrounding bone (Augustin et al. 2009; Bacci et al. 2019; Benington et al. 2002; Frösch et al. 2019; Kim et al. 2010; Lucchiari et al. 2016; Marzook et al. 2020; Mihali et al. 2018; Oh et al. 2011) or, alternatively, on the drill surface at a later time point (Augustin et al. 2009; Benington et al. 2002; Fraguas de San José et al. 2020; Oh et al. 2011; Scarano et al. 2020). This leads to a similar underestimation of the temperature rise.

In contrast, a system capable of directly recording the bone‐drill interface thermographically was introduced by Lajolo et al. (2018). In this system, bone samples were completely perforated during pilot drilling, and an infrared measuring device positioned on the opposite side recorded the point at which the drill perforated the bone. However, this approach required further refinement to accommodate extension osteotomies by preventing irrigation liquid from passing through the pilot hole before the osteotomy is started. To the best of our knowledge, the present study is the first to investigate an irrigated dental implant cavity expansion procedure using a thermographic system that measures the temperature directly at the bone‐drill interface. This approach avoids the inaccuracies caused by leaking irrigation fluid, time delays, or measurements taken from the surrounding bone.

Contact pressure is presumed to influence temperature increases during drilling; however, the literature remains divided on its effect. Some studies suggest that higher pressure leads to increased temperatures (Harder et al. 2018; Oliveira et al. 2012), while others report a reduction in temperature with increased pressure (Bogovic et al. 2016). In the present study, a surgeon applied uniform freehand drilling, calibrated to a contact pressure of 10 N using a scale, to simulate a representative clinical setting. The drilling time resulted from the contact pressure applied (10 N) and was not standardized separately. It was typically 1–2 s from the start until the lamella (6 in Figure 2) was perforated. Time may also be a relevant factor for heat stress and was short due to the shallow drilling depth of 3.5 mm (7 in Figure 2).

Macroscopic drill design may influence the temperature rise during osteotomies (Chacon et al. 2006). This study did not investigate this issue; however, serially available double twisted drills similar to previous studies (Bogovic et al. 2016; Mihali et al. 2018) are used. Microscopic surface differences have been reported at non‐worn drills (Marenzi et al. 2018) and its influence on heat generation should be the subject of further investigations.

The choice and preparation of specimens influence the actual temperatures. Several studies have simulated the human mandible using bovine ribs as a model (Allsobrook et al. 2011; Barrak et al. 2018; Benington et al. 2002; Bogovic et al. 2016; Bogovič et al. 2015; Harder et al. 2018, 2013; Hochscheidt, Shimizu, Andrighetto, Moura, et al. 2017a; Hochscheidt, Shimizu, Andrighetto, Pierezan, et al. 2017b; Lucchiari et al. 2016; Mercan et al. 2019; Mihali et al. 2018; Misic et al. 2011; Oliveira et al. 2012; Scarano et al. 2007, 2020; Strbac et al. 2014). However, other donor sites and species might also serve as suitable models (Aerssens et al. 1998). Specimens with higher bone density and greater cortical thickness are known to generate more heat during drilling (Augustin et al. 2009; Augustin, Zigman, et al. 2012b; Mihali et al. 2018). In the present study, the cortical thickness was standardized to 3.5 ± 0.01 mm, which corresponds to thick (Tanaka et al. 2018; Wang et al. 2020) or average (Song et al. 2009) human mandibular cortical bone. Since heat generation during drilling is maximal immediately basal to the cortical bone (Augustin et al. 2009; Bogovic et al. 2016; Zipprich et al. 2019), the impact of cancellous bone on temperature rise is considered minor. Especially, drilling length is not known to influence heat generation (Gehrke et al. 2018; Kapse et al. 2022) so the usage of an unconventional drilling length (4.3 mm), because of the experimental setup described above, is a minor limitation. To the best of our knowledge, this is the first study to investigate an irrigated dental implant cavity expansion procedure while minimizing donor‐related variations by standardizing cortical thickness.

The findings of this study were compared with those of previous research investigating bone temperatures during extension drilling. As the initial temperature of bone samples significantly influences the absolute measured values, the temperature increases rather than the absolute values were used for comparison to ensure consistency.

In a study by Calvo‐Guirado et al., temperature increases of drilling protocols were found to be cumulative, intensifying with each successive drilling step. According to this study, overall clinical protocols are expected to generate higher temperatures than those measured during individual osteotomies, as the implant bed does not fully cool between steps (Calvo‐Guirado et al. 2015). Calvo‐Guirado et al. reported temperature differences (∆*T*) of 6.041°C when drilling irrigated at 700 rpm (2.4–3.0 mm). In the present study, a temperature difference of 6.58°C (SS‐WI‐600rpm) was observed. While the absolute temperature increases in this study were higher, the results are broadly consistent.

Mihali et al. observed higher temperatures with smaller (3 mm) compared to larger reaming steps (3.6 mm), suggesting an inverse correlation between step size and temperature, which the present findings did not confirm (Mihali et al. 2018). However, in the present study, Group 3 (SS‐WI‐1000rpm) produced a similar increase of 8.69°C like the increase of 9.08°C in the study of Mihali et al. when drilling from 2.4 to 3 mm with irrigation and 800 rpm.

In an ex vivo study of Bacci et al. temperature changes during the reaming step from 2.8–3.5 mm performed at 500 rpm without irrigation were recorded thermographically with a pyrometer at the drill entry point (Bacci et al. 2019). The average temperature increase of 0.64°C; the average temperature increase in the present study was significantly higher at 17.79°C (SS‐NI‐600rpm). The substantially lower temperatures reported in the ex vivo study can be attributed to the measurement method, which captured temperatures at the outer bone surface rather than at the bone–drill interface. Additionally, the Dr. Meter IR‐20 pyrometer (Amazon 2021) has been shown to underestimate maximum temperatures, as previously discussed. Nevertheless, the findings of the present study align with the observed positive correlation between drill step size and temperature increase.

The present findings contradict the results of Bogovic et al., which suggested an inverse correlation between rotation speed, step size, and temperature increase (Bogovic et al. 2016).

In their study, the average temperature increase during the expansion from 2.0 to 3.0 mm at 800 rpm with irrigation was 9.26°C. In contrast, the present study reported a higher temperature increase of 13.71°C in group 6 (LS‐WI‐1000rpm). These discrepancies can be attributed to the limitations of thermocouples, which tend to underestimate maximum temperatures.

The influence of rotation speeds between 1000 and 2000 rpm on temperature development and osseointegration in dental osteotomies was analyzed using rabbit models in vivo (Marzook et al. 2020). In their study, a temperature increase of 3.54°C was observed during the irrigated expansion of a cavity from 3.0 to 3.5 mm at 1000 rpm. They described an inverse correlation between rotation speed and temperature increase. By contrast, the present study recorded a significantly higher temperature increase of 8.68°C under similar conditions (SS‐WI‐1000rpm) and finds a positive correlation between rotation speed and temperature increase.

Lucchari et al. reported temperature increases below 1°C for irrigated and non‐irrigated protocols at drill speeds ranging from 800 to 500 rpm when expanding 2.2 mm to 3.5 mm diameter cbone cavities (Lucchiari et al. 2016). This finding is likely influenced by the use of a pyrometer as already discussed. In contrast, the present study recorded significantly higher temperatures in group 2 (SS‐WI‐600rpm), with an average increase of 6.88°C.

Temperature standard deviations within groups 9 (SS‐NI‐1000rpm), 12 (LS‐NI‐1000rpm) and the preliminary test series (LS‐NI‐1000rpm) were high (SD > ± 15°C). These high variances should be investigated furthermore and have not been described in the literature yet.

The present study findings demonstrate that temperatures during implant site preparation are higher than previously assumed. In groups without irrigation, critical temperatures exceeding 47°C were observed, underscoring the necessity of irrigation to prevent thermal damage. In contrast, heat stress during osteotomies in groups 1 (SS‐WI‐200rpm) and 4 (LS‐WI‐1000rpm) remained notably low, with peak temperatures of 38.08°C (∆*T* 1.48°C) and 38.81°C (∆*T* 2.21°C). These values are classified as harmless. Therefore, short drilling sequences with substantial differences in drill diameter can be considered safe, provided rotational speeds are kept low (200 rpm) and irrigation is consistently applied.

Bone captured within the flutes of spiral drills during implant site preparation is considered a potential graft material for GBR techniques due to its biological potential (Anitua et al. 2007; Buser et al. 2008; Liang et al. 2017). Low‐speed drilling protocols without irrigation provide a higher quantity of harvested bone particles (Bernabeu‐Mira et al. 2024) and have been considered as “safe” since previous studies did not find critical temperature stress (> 47°C) (Bernabeu‐Mira et al. 2021). However, the findings of the present study suggest that low‐speed osteotomy procedures (200 rpm) without irrigation can result in significant heat stress to the bone and should therefore be avoided. Even at very low speeds (50 rpm) the newly established model predicts a probability of exceeding the temperature threshold of W = 0.60 (SS‐NI‐50rpm). The authors strongly recommend the consistent use of new, sharp drills, particularly for the final drilling step before implant placement. Additionally, bone harvesting techniques performed without irrigation should be limited to carefully selected cases with favorable conditions, such as low cortical thickness, healthy bone, low rotational speeds, intermittent drilling, minimal pressure, and small drill diameter expansions.

The duration of exposure is an important factor in the assessment of temperature‐induced damage to bone structures. The maximum amount of exposure tolerated by bone decreases with increasing temperature (Lundskog 1972). This factor was not investigated here and should be the subject of further investigation.

## 5 Conclusions

Within the limitations of this in vitro study, we can state that small and large enlargements of osteotomies from 2.4 to 3.2 mm and 2.4 to 3.8 mm do not generate any critical temperatures at low rotational speed (200 rpm) when saline irrigation is provided. However, dental osteotomies should not be performed without irrigation, even at low rotational speeds, to prevent heat‐induced damage. The potential impact of these findings on the early failure rates of immediately loaded implants requires further investigation through clinical studies.

## Author Contributions

**Karl Paeßens:** writing – original draft, writing – review and editing, methodology, visualization, investigation, conceptualization, formal analysis, software, data curation, resources, supervision, validation. **Leonard van Bebber:** conceptualization, investigation, methodology, resources, data curation. **Holger Zipprich:** conceptualization, writing – original draft, writing – review and editing, supervision, visualization, data curation, methodology, project administration, resources, validation. **Paul Weigl:** project administration, resources, supervision, validation, visualization, writing – review and editing, formal analysis.

## Disclosure

Permission to reproduce materials from other sources: No material was reproduced.

## Ethics Statement

The Promotionsausschuss Medizin Goethe Universität waived the need for approval because the bovine bones for the study were obtained from a slaughterhouse.

## Consent

The authors have nothing to report.

## Conflicts of Interest

The authors declare no conflicts of interest.

## Data Availability

The data that support the findings of this study are openly available in figshare at https://figshare.com/s/728489599493d7bfbf20 and the reference number will be delivered once published.
